# Relating Measurement Invariance, Cross-Level Invariance, and Multilevel Reliability

**DOI:** 10.3389/fpsyg.2017.01640

**Published:** 2017-10-10

**Authors:** Suzanne Jak, Terrence D. Jorgensen

**Affiliations:** Research Institute of Child Development and Education, University of Amsterdam, Amsterdam, Netherlands

**Keywords:** measurement invariance, multilevel structural equation modeling, multilevel confirmatory factor analysis, cross-level invariance, multilevel reliability

## Abstract

Data often have a nested, multilevel structure, for example when data are collected from children in classrooms. This kind of data complicate the evaluation of reliability and measurement invariance, because several properties can be evaluated at both the individual level and the cluster level, as well as across levels. For example, cross-level invariance implies equal factor loadings across levels, which is needed to give latent variables at the two levels a similar interpretation. Reliability at a specific level refers to the ratio of true score variance over total variance at that level. This paper aims to shine light on the relation between reliability, cross-level invariance, and strong factorial invariance across clusters in multilevel data. Specifically, we will illustrate how strong factorial invariance across clusters implies cross-level invariance and perfect reliability at the between level in multilevel factor models.

## Introduction

Multilevel data are data with a clustered structure, for instance data of children clustered in classrooms, or data of employees clustered in teams. Taking data of children in classes as an example, we can distinguish two levels in the data: we denote the child level the “within level”, and the class level the “between level”. Children in the same class share class-level characteristics, such as the teacher, classroom composition, and class size. Such class-level characteristics may affect child-level variables, leading to structural differences between the responses of children from different classes. With multilevel structural equation modeling (multilevel SEM), such differences are accommodated by specifying models (such as factor models) at the different levels of multilevel data. Multilevel SEM is increasingly applied in various fields such as psychology and education.

Researchers commonly interpret standardized parameter estimates, which may lead to interpretational difficulties in multilevel models. The most common standardized solution in multilevel factor models is the level-specific standardization (Hox, 2010). This type of standardization involves standardizing the within-level parameter estimates with respect to the within-level variance, and standardizing the between-level parameter estimates with respect to the between-level variance. In this standardization, it is common to find very high correlations among between-level factors, and to find standardized factor loadings that are (almost) one at the between level (e.g., Hanges and Dickson, 2006; Bakker et al., 2015). The reason that these findings are common is that residual variance at the between level is often (close to) zero (Hox, 2010), leading to relatively high standardized between-level factor loadings. At the same time, the *unstandardized* between-level factor loadings may not differ from the factor loadings at the within level. However, researchers tend to interpret the larger standardized parameter estimates at the between level as if the construct meaning is very different across the two levels of the analyses. For example, Whitton and Fletcher (2014) found larger standardized between-level factor loadings than within-level factor loadings, and concluded that the measured construct is a “group-level construct,” and that future research should emphasize interpretation at the group level rather than on the individual level. However, in the same article they reported the intraclass correlations for the subscales, showing that only 38% of the variance was at the between level, while 62% of the variance was at the individual level.

The current article explains and illustrates that neither the (near) absence of residual variance at the between level (with consequently high standardized factor loadings at the between level) nor very high reliability at the between level should be interpreted as different factors operating at the within and between level. In the next three paragraphs we briefly introduce the three concepts of measurement invariance across groups (or clusters), invariance across levels in multilevel SEM, and reliability in multilevel SEM. The goal of this article is to illuminate the relations between these three issues. Therefore, in section Relations between the three concepts we discuss each combination of concepts, and in section Example we provide illustrations with real data from students nested within schools.

### Measurement invariance across groups

Testing for measurement invariance is important to evaluate whether items measure the same attributes for different (groups of) respondents (Mellenbergh, 1989; Meredith, 1993). For example, if the items in a mathematical ability test measures the same attribute in boys and girls, then boys and girls with equal mathematical ability should, on average, have identical observed scores. That is, mean differences in observed scores should reflect mean differences in the true mathematical ability scores. If this is not the case, there is measurement bias. For example, given equal mathematical ability, a specific item with a worded math problem may be easier to solve for girls, because girls are generally better in reading than boys (Wei et al., 2012). For that reason, given equal levels of mathematical ability, girls might have more correct answers on this item than boys would. The item is therefore biased with respect to gender.

Structural equation modeling (SEM) with latent variables provides a flexible method to test for measurement invariance. When measurement invariance is tested with respect to a grouping variable (e.g., boys vs. girls), we can use multigroup factor analysis (MGFA) with structured means (Sörbom, 1974). In the multigroup method, specific manifestations of measurement bias can be investigated by testing across-group constraints on intercepts and factor loadings. Adequate comparisons of factor means across groups are possible if strong factorial invariance across groups holds (Meredith, 1993; Widaman and Reise, 1997). Strong factorial invariance across groups comprises equality of factor loadings and intercepts across groups. The model for the observed variables' means and covariances in group *j* under strong factorial invariance across groups will therefore be:

(1) $$\begin{array}{r} {\text{μ}}_{j}=\text{ν}+{\Lambda \kappa }_{j},\text{and} \end{array}$$

(2) $$\begin{array}{r} {\text{Σ}}_{j}={\Lambda \Phi }_{j}{\text{Λ}}^{\prime }+{\text{Θ}}_{j}, \end{array}$$

where **μ**_*j*_ and **Σ**_*j*_ represent respectively the mean vector and covariance matrix of the observed variables in group *j*, **κ**_*j*_ and **Φ**_*j*_ represent respectively the vector of common factor means and the covariance matrix of the common factors in group *j*, **Θ**_*j*_ is the matrix with residual (co)variances of observed variables in group *j*, **ν** is a vector of intercepts [interpretable as the means of the residual factors, Meredith and Teresi (2006)] that is invariant across groups, and **Λ** is a matrix with factor loadings (regression coefficients relating the common factor to the factor indicators) that is also invariant across groups. These equations show that if strong factorial invariance holds, differences in observed means across groups (**μ**_*j*_), are a function of differences in factor means across groups (**κ**_*j*_), because nothing else on the righthand side of Equation (1) varies across groups. Also, note that the matrix with factor loadings is part of the model for the means as well as the model for the covariances. In order to provide scale and origin to the common factors, factor means and variances have to be fixed to some value in one reference group (commonly 0 for the factor means and 1 for the factor variances), and can be freely estimated in all other groups.

If the intercepts differ across groups, but the factor loadings are invariant, then strong factorial invariance is rejected, but weak factorial invariance holds. Group differences in intercepts are called “uniform bias” and differences in factor loadings are called “non-uniform bias” (Millsap and Everson, 1993).

### Invariance across levels in two-level SEM

Multilevel SEM is a useful statistical technique to analyze data from many different groups, such as data from children in different school classes. Multilevel SEM then allows researchers to separate the levels of analysis (Muthén, 1990; Rabe-Hesketh et al., 2004). For example, one could evaluate differences in the students' average mathematical ability across different school classes (called the between level) and separately evaluate differences in students' relative mathematical ability within their class (called the within level). In two-level SEM, the vector of continuous response variables **y**_*ij*_, is split into a vector of cluster means (**μ**_*j*_), and a vector of individual deviations from the respective cluster means (**η**_*ij*_ = **y**_*ij*_ − **μ**_*j*_):

(3) $$\begin{array}{r} {\text{y}}_{ij}={\text{μ}}_{j}+{\text{η}}_{ij}. \end{array}$$

It is assumed that **μ**_*j*_ and **η**_*ij*_ are independent. The covariances of **y**_*ij*_ (**Σ**_TOTAL_) can be written as the sum of the covariances of **μ**_*j*_ (**Σ**_BETWEEN_) and the covariances of **η**_*ij*_ (**Σ**_WITHIN_):

(4) $$\begin{array}{r} {\text{Σ}}_{\text{TOTAL}}={\text{Σ}}_{\text{BETWEEN}}+{\text{Σ}}_{\text{WITHIN}} \end{array}$$

The within-level and between-level covariances are modeled simultaneously but independently (unless across-level constraints are applied). For example, we may consider a two-level factor model for *p* observed variables and *k* common factors at each level:

(5) $$\begin{array}{r} {\text{Σ}}_{\text{BETWEEN}}={\text{Λ}}_{\text{BETWEEN}}{\text{Φ}}_{\text{BETWEEN}}{\text{Λ}}_{\text{BETWEEN}}^{\prime }+{\text{Θ}}_{\text{BETWEEN}},\\ {\text{Σ}}_{\text{WITHIN}}={\text{Λ}}_{\text{WITHIN}}{\text{Φ}}_{\text{WITHIN}}{\text{Λ}}_{\text{WITHIN}}^{\prime }+{\text{Θ}}_{\text{WITHIN}}, \end{array}$$

where **Φ**_BETWEEN_ and **Φ**_WITHIN_ are *k* × *k* covariance matrices of common factors, **Θ**_BETWEEN_ and **Θ**_WITHIN_ are *p* × *p* (typically diagonal) matrices with residual (co)variances, and **Λ**_BETWEEN_ and **Λ**_WITHIN_ are *p* × *k* matrices with factor loadings at the between and within level, respectively.

In principle, the factor structures at the two levels can be completely different. However, in many situations the results are hard to interpret without assuming some constraints across levels. Stapleton et al. (2016) provide a nice overview of types of constructs in multilevel models. They showed that if the between-level construct represents the aggregate of the characteristics of individuals within the clusters, cross-level constraints are required. Specifically, to correctly model such constructs, the same factor structure has to apply to both levels, and factor loadings should be equal across levels. In cross-cultural research, equality of factor loadings across levels is called isomorphism (Tay et al., 2014). Across-level invariance ensures that the factors at different levels can be interpreted as the within-level and between-level components of the same latent variable (van de Vijver and Poortinga, 2002). This decomposition also allows for free estimation of the factor variance at the between level, and consequently for the calculation of the factor intraclass correlation (Mehta and Neale, 2005), representing the percentage of factor variance at the between level.

### Reliability in multilevel factor models

Lord and Novick (1968) defined reliability as the squared correlation between true and observed scores. An alternative (but mathematically equivalent) definition of reliability is that it is the ratio of the true score variance over the total variance (e.g., McDonald, 1999). The “true score variance” in this definition points to the part of the total score variance that is free from random error. Assuming that one has access to the true score variance, the reliability is:

(6) $$\begin{array}{r} \frac{Var\left(T\right)}{Var\left(T\right)+Var\left(E\right)} \end{array}$$

where Var(T) is the true score variance, and Var(E) is measurement error variance.

In factor models, the common factor variance is used as an estimate of the true score variance. The remaining variance in an indicator stems from a residual factor (**δ**) that consists of two components: a reliable component, **s**, which is a stable component over persons, but not shared with other indicators; and a truly random component, **e** (Bollen, 1989). One difference between the concept of reliability in classical test theory (CTT) and the concept of reliability in the factor modeling framework is that in CTT, the variance of the stable component **s** is part of the reliable variance (included in the nominator in Equation 6), whereas in the factor analysis framework it is considered an unreliable part (only included in the denominator in Equation 6)^1^.

The common factor therefore represents the reliable *common* parts of the indicators. In the SEM definition of reliability (Bollen, 1989, p. 221), the regressions of the indicator variables on the common factors represent the systematic components of the indicators, and all else represents error. The reliability of a single indicator can therefore be evaluated based on the size of the factor loading. Indices that focus on the reliability of scales with multiple indicators commonly represent some form of the ratio of common indicator variance over total indicator variance.

Geldhof et al. (2014) provided an overview of reliability estimation in multilevel factor models. They showed that level-specific reliability estimates are preferable to single-level reliability estimates when the variance at the between level is substantial. Also, they found that estimated between-cluster composite reliability (ω) was generally more unbiased than between-cluster alpha (α) and maximal reliability estimates. In this article we will therefore focus on composite reliability. Composite reliability in a congeneric factor model is defined as the ratio of *common* indicator variance over the *total* indicator variance (Werts et al., 1974; Raykov, 1997). Assuming no covariances between residual factors, and no cross loadings, composite reliability of a scale with factor variance φ, factor loadings λ_1_, λ_2_, …, λ_*k*_ and residual variances θ_1_, θ_2_, …, θ_*k*_ can be estimated by:

(7) $$\begin{array}{r} \omega =\;\frac{{\left(\sum_{i=1}^{k}\lambda_{i}\right)}^{2}\varphi }{{\left(\sum_{i=1}^{k}\lambda_{i}\right)}^{2}\varphi +\sum_{i=1}^{k}\theta_{i}} \end{array}$$

Level-specific composite reliability is estimated by plugging in the level-specific factor loading and residual variance estimates into the formula for ω. Cluster-level reliability as estimated with Equation (7) reflects the degree to which group-level differences in a researcher's observed data can be generalized to represent between-group differences in a construct of interest (Geldhof et al., 2014).

## Relations between the three concepts

### How invariance between groups relates to between-level reliability

Given that in factor analysis the reliable part of the indicator is the part that reflects the common factor, reliable mean differences in observed variables between groups would reflect mean differences in common factors across groups. Lubke et al. (2003) very nicely explained the relationship between sources of within- and between-group differences and measurement invariance in the common factor model. They explicated that measurement invariance implies between-group differences cannot be due to other factors than those accounting for within-group differences.

Suppose observed mean differences between groups are due to entirely different factors than those that account for the individual differences within a group. The notion of “different factors” as opposed to “same factors” implies that the relation of observed variables and underlying factors is different in the model for the means as compared with the model for the covariances, that is, the pattern of factor loadings is different for the two parts of the model. If the loadings were the same, the factors would have the same interpretation. In terms of the multigroup model, different loadings imply that the matrix **Λ** in Equation (1) differs from the matrix **Λ** in Equation (2) (Equation numbers adjusted). However, this is not the case in the MI (measurement invariance) model. Mean differences are modeled with the same loadings as the covariances. Hence, this model is inconsistent with a situation in which between-group differences are due to entirely different factors than within-group differences (Lubke et al., 2003, p. 552).

In other words, if measurement invariance holds, then observed mean differences between groups reflect differences in the means of common factors across groups. Suppose for example that one has used several indicators to measure mathematical ability in boys and girls. Within the group of boys, the mathematical ability likely differs from boy to boy, leading to differences in the observed indicators. Similarly, within the group of girls there will be systematic differences between girls that are caused by individual differences in mathematical ability. In addition, the mean mathematical ability may differ between boys and girls. If measurement invariance holds, all group mean differences in the observed scores are caused by differences in the mean mathematical ability across groups. If the differences within and between groups are due to entirely different factors, or if there are additional factors besides mathematical ability affecting the between-group scores, then measurement invariance does not hold (Lubke et al., 2003). In this case, the measurement of between-group differences is not reliable, because differences between groups do not only reflect differences in common factors across groups.

### How invariance between groups relates to invariance across levels

When researchers are interested in differences between large numbers of groups, it becomes infeasible to conduct multigroup modeling. In these cases it is sensible to treat group as a random rather than a fixed variable, and to use multilevel techniques (Muthén and Asparouhov, 2017). For example, if a researcher wants to evaluate differences in latent variables between many countries, one could use a two-level model in which countries are treated as the clustering variable (Jak, 2017). In this example, the between-level model would represent country-level mean differences in the variables, and the within-level model would represent differences in individual deviations from the respective country means. Jak et al. (2013, 2014) provided a short overview of how three increasingly restrictive assumptions across *groups/clusters* (configural, weak, and strong factorial invariance) lead to testable restrictions across *levels* in a two-level. Specifically, they showed how weak factorial invariance across groups in a multigroup factor model translates to equal factor loadings across levels in a two-level factor model (Equations 9 and 10 in Jak et al., 2013). When strong factorial invariance holds, in addition to equal factor loadings across levels, the residual variance at the between level is zero (Equation 11 in Jak et al., 2013). We provide a more detailed and annotated derivation of these models in Appendix A in Supplementary Material. The first two columns in Table 1 provide an overview of restrictions in a multigroup model, and the implications for a two-level model.

**Table 1:** Comparison of the restrictions in a multigroup model and the implications in a two-level model with different levels of factorial invariance.

	**Restrictions in multigroup model**	**Implications in two-level model**	**Implications reliability**
**LEVEL OF FACTORIAL INVARIANCE**			
Configural	pattern(**Λ**_g_) = pattern(**Λ**)	**–**	
Weak	**Λ**_g_ = **Λ**	**Λ**_WITHIN_ = **Λ**_BETWEEN_	
Strong	**Λ**_g_ = **Λ**, **ν**_g_ = **ν**	**Λ**_WITHIN_ = **Λ**_BETWEEN_, **Θ**_BETWEEN_ = 0	**ω**_BETWEEN_ = 1

***ν** is a p-dimensional vector of intercepts. Subscript g is used for group/cluster*.

### How invariance across levels relates to reliability

In principle, level-specific reliability estimates can be calculated using the estimates of a two-level factor model without cross-level invariance constraints. However, in that case, the interpretation of the common factor at the two levels is not identical. In practice, research questions will often be answered using multilevel data that involves what Stapleton et al. call “configural constructs.” These are constructs for which the interest is both in the within and between cluster differences, and the between-level construct represents the aggregate of the within-level characteristics. Examples are evaluation of differences in citizenship behavior within and between countries (Davidov et al., 2016) and the evaluation of teacher-student relationship quality within and between school classes (Spilt et al., 2012). These types of models require cross-level invariance restrictions on the factor loadings. When using Equation (7) to estimate composite reliability at the both levels in such a model, and provided that the two-level factor model with cross-level invariance fits the data satisfactorily, one would plug in the same unstandardized factor loadings when calculating within-level and between-level composite reliability. However, the factor variances and residual variance likely differ across levels, leading to different reliability estimates at the two levels. In the case that cluster invariance holds for all items, all residual variances at the between level will be zero, leading to perfect composite reliability at the between level (as indicated in the last column of Table 1). In practice, it is unlikely to find cluster invariance for *all* items, as it is unlikely that strong factorial invariance across clusters holds for *all* items. Perfect composite reliability is therefore expected to be rare in practice. Often, researchers find partial strong factorial invariance across groups (Byrne et al., 1989). Similarly, it is quite common to find perfect reliability for *some* of the items at the between level (e.g., Bottoni, 2016; Zee et al., 2016).

## Example

### Data

We illustrate the multigroup modeling, two-level modeling, and multilevel reliability analysis using six items to measure “emotional well-being” that were included in round 2012 of the European Social Survey (Huppert et al., 2009; ESS Round 6: European Social Survey, 2014). Three items are positively formulated, asking how often in the last week a respondent was happy (WRHPP), enjoyed life (ENJLF), and felt calm and peaceful (FLTPCFL). The other three items were negatively phrased, asking how often in the last week a respondent felt depressed (FLTDP), felt sad (FLTSD), and felt anxious (FLTANX). The items were scored on a 4-point scale ranging from 0 (*none or almost none of the time*) to 3 (*all or almost all of the time*). Round 2012 of the ESS included data from 54,673 respondents from 29 countries on these items.

### Analysis

All models were fit to the data with M*plus* version 7 (Muthén and Muthén, 1998–2015), using maximum likelihood estimation (MLR). This estimation method provides a test statistic that is asymptotically equivalent to the Yuan–Bentler T2 test statistic (Yuan and Bentler, 2000), and standard errors that are robust for non-normality. For illustrative purposes, we treat the responses to the 4-point scale as approximately continuous.

Statistical significance of the χ^2^ statistic (using α = 0.05) indicates that exact fit of the model has to be rejected. With large sample sizes, very small model misspecifications may lead to rejection of the model. Therefore, we also consider measures of approximate fit; the root mean square error of approximation (RMSEA; Steiger and Lind, 1980) and the comparative fit index (CFI; Bentler, 1990). RMSEA values smaller than 0.05 indicate close fit, and values smaller than .08 are considered satisfactory (Browne and Cudeck, 1992). CFI values over 0.95 indicate reasonably good fit (Hu and Bentler, 1999). In addition, for model comparison we evaluate the BIC (Raftery, 1986, 1995), of which smaller values indicate better fit.

Emotional well-being is an individual-level construct, of which the aggregated scores at the country level may differ. In the terminology of Stapleton et al. (2016), this is a configural construct, which needs cross-level equality constraints on the factor loadings.

### Measurement model

First, we fitted a two factor model to the well-being items on the merged dataset of all countries. The fit of this model was satisfactory, $\chi_{\left(8\right)}^{2}$ = 2633.591, *p* < 0.05, RMSEA = 0.078, 90% CI [0.075; 0.080], CFI = 0.98. Inspection of modification indices showed that the modification index of a cross loading of FLTPCFL on the factor Negative well-being was around three times larger than the other modification indices. This item is the only positively phrased item that refers to feelings, while all negatively phrased items refer to feelings. Therefore, we decided to add this (negative) cross loading. The resulting model fitted the data satisfactorily, $\chi_{\left(7\right)}^{2}$ = 1352.814, RMSEA = 0.059, 90% CI [0.057; 0.062], CFI = 0.99, and was considered the final measurement model^2^.

### Multigroup model

Next we fitted the three multigroup models representing configural invariance, weak factorial invariance, and strong factorial invariance to the data of 29 countries, with Albania as the reference country. The fit results of these three models can be seen in Table 2. Overall fit of the models with configural and weak factorial invariance can be considered satisfactory, but strong factorial invariance does not hold according to all fit indices. In addition, the model with weak factorial invariance has the lowest BIC-value. Apparently, at least some intercepts were not invariant across countries. Rejection of strong factorial invariance can be caused by relatively large differences in intercepts across a few countries, relatively small differences in intercepts across many countries, or a combination of both. In order to find out which items were most biased, we counted the number of countries in which each item's intercept had a high modification index. Table 3 shows the number of countries for which specific items were flagged to be biased based on whether an intercept's modification index exceeded a threshold of 50 or 100. Based on these counts, the item FLTANX seems to be most biased, and the item FLTSD seems the least biased.

**Table 2:** Model fit of three increasingly restrictive multigroup invariance models on the well-being items.

	***df***	**χ^2^**	**RMSEA [90%CI]**	**CFI**	**BIC**
Configural invariance	203	1742.848	0.063 [0.061; 0.066]	0.985	637061.39
Weak factorial invariance	343	3168.430	0.066 [0.064; 0.068]	0.972	636959.90
Strong factorial invariance	455	12471.471	0.118 [0.117; 0.120]	0.882	645041.28

**Table 3:** Number of countries with a modification index of the intercept >50 and >100 per item.

	**#MI > 50**	**#MI > 100**
WRHPPY	9	4
ENJLF	13	5
FLTPCFL	10	8
FLTDPR	13	7
FLTSD	8	2
FLTANX	18	14

*#MI = number of modification indices*.

### Two-level model

We fitted three increasingly restrictive two-level models. The fit results can be found in Table 4. The first model is a two-level model specifying the measurement at the within and between levels without any constraints across levels. The fit of this model was satisfactory according to the RMSEA and CFI. However, this model does not allow for a meaningful interpretation of the factors at the two levels. Next, we constrained the factor loadings to be equal across levels, and freely estimated the factor variances at the between level. This model fitted the data significantly worse, which may be expected given the large sample size, but lead to a lower BIC-value. The overall fit was still acceptable according to the RMSEA and CFI.

**Table 4:** Model fit of three increasingly restrictive two-level models on the well-being items.

	***df***	**χ^2^**	**RMSEA**	**CFI**	**BIC**
Two-level CFA	14	516.692	0.026	0.976	641634.92
Cross-level invariance	19	619.519	0.024	0.972	641597.23
Strong factorial invariance	25	6880.934	0.071	0.679	647276.03

Constraining the loadings to equality across levels allows computation of the factor ICC. For positive well-being, the ICC was 0.06/(1 + 0.06) = 0.057, indicating that 5.7% of the factor variance was on the country level, and for negative well-being the ICC was 0.133/(1 + 0.133) = 0.117, indicating that 11.7% of the factor variance was on the country level.

The model assuming strong factorial invariance, that is, the model with the between-level residual variances fixed to zero, fitted the data much worse than the first two models based on all fit indices, indicating that strong factorial invariance does not hold across countries. This finding matches the conclusion from the multigroup analysis. Non-zero residual variance at the between level shows that there are other factors than well-being influencing the country level scores on the items. Table 5 shows the modification indices for each item's residual variance, and the actual decrease in χ^2^ when freeing each item's residual variances. It is notable that, similar to the analysis of Muthén and Asparouhov (2017), the modification indices are not a good approximation of the actual drop in χ^2^ when freeing the respective parameter. However, the ordering of the amount of bias present in each item is identical for the two methods. The item FLTANX seems to have the most bias, and the item FLTSD seems to be the least biased. These findings match the results from the multigroup analysis.

**Table 5:** Modification indices (MIs) and chi-squared differences for releasing specific residual variances.

		**free θ_i_**
**Item**	**MI**	**Δχ^2^**
WRHPPY	8895.463	661.022
ENJLF	28777.092	1229.299
FLTPCFL	40919.137	1410.159
FLTDPR	36531.309	1380.276
FLTSD	8491.897	641.51
FLTANX	147722.922	2868.184

**Figure 1:**
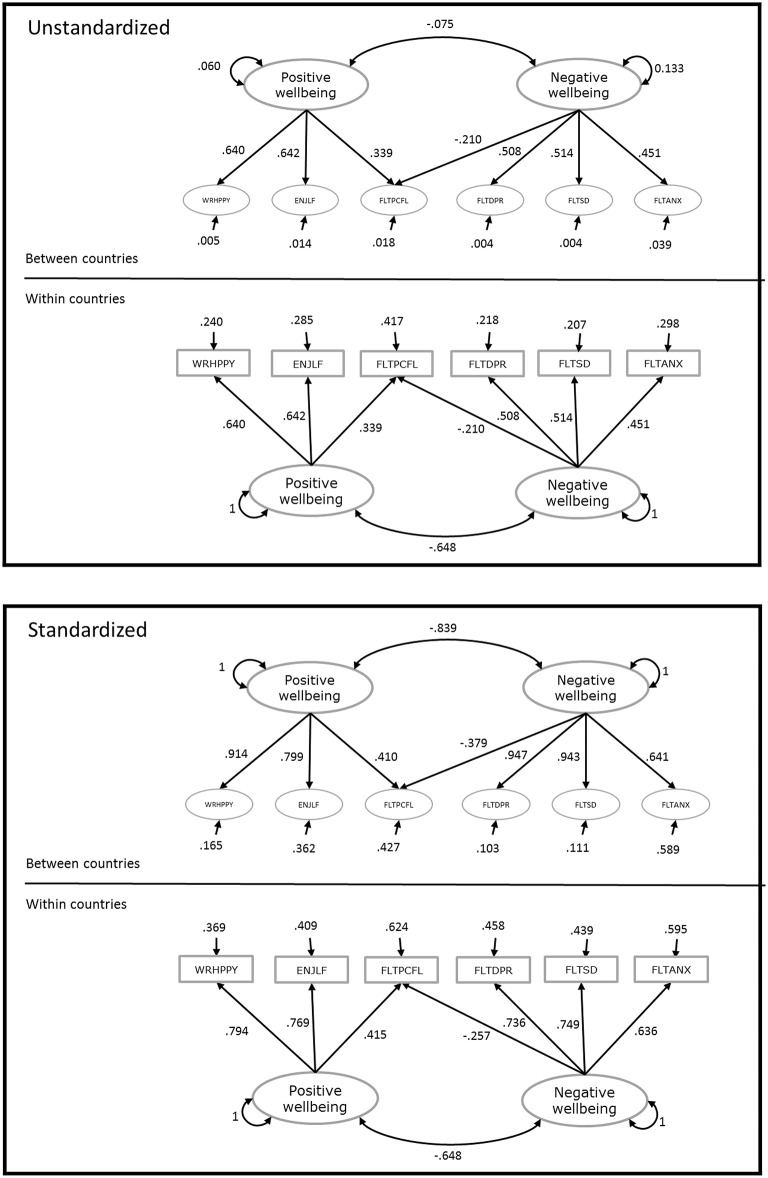
Unstandardized and standardized parameter estimates from the two-level model with cross-level invariance.

Figure 1 shows the unstandardized and standardized parameter estimates from the two-level model with cross-level invariance. It can be seen that although the factor loadings are constrained across levels, the standardized factor loadings are different across levels, and they are quite high at the between level, specifically for the least biased indicators. Assuming the model is configured correctly (i.e., the same construct operates at the individual and country levels), the standardized residual variance at the between level represents the proportion of item variance at the country level that is not explained by the common factor(s). These proportions are highest for the items FLTANX and FLTPCFL, and smallest for the item FLTSD, which again matches the previous conclusions about which items are most biased across countries.

### Reliability

We used a two factor model with a cross loading as the measurement model. However, the formula for composite reliability that we presented (Equation 7) is only suited for congeneric factor models. Raykov and Shrout (2002) provided a method to obtain estimates of reliability for composites of measures with non-congeneric structure. Treating well-being as a multidimensional construct at each level, composite reliability for the six items was estimated as 0.77 at the within level, and 0.87 at the between level. As expected, the reliability at the between level is much higher than at the within level. The indicators that contribute most to the composite reliability estimates are the indicators with the largest standardized factor loadings (and least residual variance). For the positive well-being scale, the most reliable indicator at the between level is WRHPPY, and for the negative well-being scale the most reliable indicators are FLTDPR and FLTSD. These two items are also the items that came out as least biased in the multigroup analysis, as well as in the two-level analysis. The item with the lowest between-level standardized factor loadings is FLTPCFL, which loads on both the positive and the negative well-being factor. However, for items that load on multiple common factors, we cannot take the individual standardized factor loadings as direct indications of unbiasedness, because it does not take into account the amount of variance that is explained by the other factor(s).

## Discussion

The goal of our paper was to elucidate the relationship between measurement invariance across clusters, loading invariance across levels, and reliability in multilevel SEM. We used a real-data example to illustrate special issues that applied researchers should consider, which we summarize below. Invariance of loadings across levels is implied for configural constructs, so testing equality constraints on loadings across levels constitutes a test of whether a between-level construct can be interpreted as an aggregate of its within-level counterpart. Invariance of loadings across levels is also implied when factor loadings are assumed to be equal across clusters (i.e., when weak factorial invariance across clusters holds). Cross-level invariance is a necessary but not sufficient condition for weak factorial invariance across clusters. This means that if a construct cannot be regarded as configural (i.e., if cross-level invariance does not hold), then weak factorial invariance across clusters does not hold. But the reverse does not hold: If a construct *is* configural, that does not necessarily imply that weak factorial invariance across clusters also holds, because non-uniform bias across clusters has also been found to show up as residual variance at the between level (Jak et al., 2013). To summarize, equal factor loadings across clusters imply equal factor loadings across levels (and thus a configural construct), but not the other way around^3^.

Equality of intercepts, on the other hand, cannot be tested across levels because the intercepts apply only to the observed variables, not separately for within- and between-level components. The common practice of fixing factor means to zero for identification of the mean structure makes it easy to show that within-level intercepts are expected to be zero. This is because the within-level component (**η**_*ij*_) of **y**_*ij*_ is partitioned from the group means (**μ**_*j*_), which are the between-level components of **y**_*ij*_. Thus, as shown in the Appendix in Supplementary Material, the group means of **y**_*ij*_ are a function of **τ**_*j*_ because their between-level components **μ**_*j*_ are themselves a function of **τ**_*j*_. Strong invariance can, however, be tested across clusters. If intercepts do not vary across clusters, that implies no between-level residual variance, so strong invariance across clusters can be tested by constraining between-level residual variances to zero in a model with cross-level loading invariance.

Finally, when working with multilevel data, reliability should be estimated separately for each level of measurement (Geldhof et al., 2014). When the construct is meant to be interpreted only at the within or between level, reliability need only be calculated at the level of interest, and a saturated model should be specified at the other level (Stapleton et al., 2016). Level-specific reliability can be interpreted for configural constructs that have analogous interpretations at each level of measurement. For example, within-level composite reliability is the proportion of variance between individuals within clusters (i.e., variability around cluster means) that is accounted for by individual differences on the within-level construct. Between-level composite reliability is the proportion of variance in cluster means that is accounted for by differences in cluster means of the same construct. Greater between-level than within-level reliability should not be mistaken for indicating that the construct has a different meaning at the between level, because (near) perfect between-level reliability (and therefore nearly zero between-level residual variance) is necessarily implied by (near) strong invariance across clusters.

## Author contributions

SJ conceptualized and designed the study, SJ selected the example data and performed the analyses, TJ critically reviewed the analyses, TJ and SJ drafted the manuscript.

### Conflict of interest statement

The authors declare that the research was conducted in the absence of any commercial or financial relationships that could be construed as a potential conflict of interest.

## Footnotes

^1^The specific variance of a measure is typically not known. In a factor model, specific variance is part of the residual variance and really only included in the denominator of Equation (6). In CTT-measures of reliability however, the specific variance may only *partly* be included in the numerator. See Bollen (1989, p. 219) for a discussion.

^2^The reported fit measures are obtained from an overall analysis on the merged dataset while ignoring the dependency of individuals within countries. Using an analysis with corrected fit statistic (Type = Complex in Mplus) leads to better model fit and similar conclusions, with $\chi_{\left(8\right)}^{2}=327.979$, *p* < 0.05, RMSEA = 0.027, 90% CI [0.025; 0.030], CFI = 0.99 for the first model, and $\chi_{\left(7\right)}^{2}=212.285$, *p* < 0.05, RMSEA = 0.023, 90% CI [0.021; 0.026], CFI = 0.99 for the modified (final) model.

^3^Testing equality of factor loadings across clusters in a multilevel framework requires estimating each loading as a random slope, represented as a Level-2 factor with freely estimated variance. Testing whether a variance equals zero would constitute a test of invariance of loadings across clusters. This topic is beyond the scope of our paper but is discussed in Kim et al. (2017) and Muthén and Asparouhov (2017).
