# Supplementary material for: Relating Measurement Invariance, Cross-Level Invariance, and Multilevel Reliability
Source: Front Psychol. 2017 Oct 10;8:1640. doi: 10.3389/fpsyg.2017.01640 (PMC5641393; doi:10.3389/fpsyg.2017.01640)
Supplement: Supplementary file 1 [file DataSheet1.pdf]

Appendix A.

Below we show how to arrive from the equations for the multigroup model to the expressions for the two-level model in smaller steps as shown in Jak, Oort, and Dolan (2013), which we refer to as JOD.

Explanation of symbols:

$\mathbf{y}_{ij}$ vector of observed scores of person i in group j

$\boldsymbol{\mu}_j$ vector of group means of observed scores in group j

$\boldsymbol{\eta}_{ij}$ vector of individual deviations from the group means for individual i in group j

$\boldsymbol{\tau}_j$ vector of group specific intercepts (residual means)

$\boldsymbol{\Lambda}_j$ matrix with group specific factor loadings

ξ_{ij} vector with factor scores of individual i in group j

$\boldsymbol{\varepsilon}_{ij}$ vector with residual factor scores of individual i in group j

$\boldsymbol{\kappa}_j$ vector with mean factor scores in group j

The observed scores of person i in group j can be decomposed in the group mean scores, $\boldsymbol{\mu}_j$, and the individual deviations from the group mean scores, $\boldsymbol{\eta}_{ij}$. So:

$$\mathbf{y}_{ij} = \boldsymbol{\mu}_j + \boldsymbol{\eta}_{ij} \quad (1)$$

or equivalently rewritten as:

$$\boldsymbol{\eta}_{ij} = \mathbf{y}_{ij} - \boldsymbol{\mu}_j \quad (2)$$

If the same factor structure holds across groups, but the values of the factor loadings differ across groups (so, if configural invariance holds), the factor model for $\mathbf{y}_{ij}$ is:

$$\mathbf{y}_{ij} = \boldsymbol{\tau}_j + \boldsymbol{\Lambda}_j \xi_{ij} + \boldsymbol{\varepsilon}_{ij} \quad \text{(Equation 4 in JOD)} \quad (3)$$

In this case, there is no elegant two-level structure, as shown by the long ugly equations 7 and 8 in JOD.

Assuming weak factorial invariance across clusters

If the values of factor loadings *are* equal across groups, so that weak factorial invariance holds, then the model for the observed scores becomes:

$$\mathbf{y}_{ij} = \boldsymbol{\tau}_j + \boldsymbol{\Lambda} \xi_{ij} + \boldsymbol{\varepsilon}_{ij} \quad \text{(subscript to } \boldsymbol{\Lambda} \text{ disappears)} \quad (4)$$

The group mean scores $\boldsymbol{\mu}_j$ can then be expressed as the group-expected value of the observed scores:

$$\boldsymbol{\mu}_j = E(\boldsymbol{\tau}_j + \boldsymbol{\Lambda} \xi_{ij} + \boldsymbol{\varepsilon}_{ij}) \quad (5)$$

The expected value of the residual $\boldsymbol{\varepsilon}_{ij}$ is zero by assumption (Bollen, 1989), therefore:

$$\boldsymbol{\mu}_j = E(\boldsymbol{\tau}_j + \boldsymbol{\Lambda} \xi_{ij}) \quad (6)$$

The expected value of the factor score within groups is the factor group mean, indicated by $\boldsymbol{\kappa}_j$, and the intercept $\boldsymbol{\tau}_j$ is a constant so:

$$\boldsymbol{\mu}_j = \boldsymbol{\tau}_j + \boldsymbol{\Lambda}\,\boldsymbol{\kappa}_j \qquad (7)$$

The individual deviations from the group means, $\boldsymbol{\eta}_{ij}$, are equal to (Eq. 2):

$$\boldsymbol{\eta}_{ij} = \mathbf{y}_{ij} - \boldsymbol{\mu}_j \qquad (8)$$

Filling in the previous expression (Eq. 7) for $\boldsymbol{\mu}_j$, we get:

$$\boldsymbol{\eta}_{ij} = \mathbf{y}_{ij} - (\boldsymbol{\tau}_j + \boldsymbol{\Lambda}\,\boldsymbol{\kappa}_j) \qquad (9)$$

Also filling in the expression for $\mathbf{y}_{ij}$ (Eq. 4) gives:

$$\boldsymbol{\eta}_{ij} = (\boldsymbol{\tau}_j + \boldsymbol{\Lambda}\,\xi_{ij} + \boldsymbol{\varepsilon}_{ij}) - (\boldsymbol{\tau}_j + \boldsymbol{\Lambda}\,\boldsymbol{\kappa}_j) \qquad (10)$$

Which simplifies (the intercepts cross out) to:

$$\boldsymbol{\eta}_{ij} = \boldsymbol{\Lambda}\,\xi_{ij} - \boldsymbol{\Lambda}\,\boldsymbol{\kappa}_j + \boldsymbol{\varepsilon}_{ij} \qquad (11)$$ [1]

Now we have expressions for the group means, and for the individual deviations from the group means. Note that the factor loading matrix $\boldsymbol{\Lambda}$ is featured in both these expressions. The model-implied covariance matrices at the within and between levels are:

$$\boldsymbol{\Sigma}_W = \mathrm{COV}(\boldsymbol{\eta}_{ij}, \boldsymbol{\eta}_{ij}) \text{ and } \boldsymbol{\Sigma}_B = \mathrm{COV}(\boldsymbol{\mu}_j, \boldsymbol{\mu}_j) \quad \text{(Eq. 3 from JOD)} \qquad (12)$$

To start with the within-level, using covariance algebra, we obtain:

$$\boldsymbol{\Sigma}_W = \mathrm{COV}(\boldsymbol{\eta}_{ij}, \boldsymbol{\eta}_{ij}), \qquad (13)$$

$$= \mathrm{COV}(\boldsymbol{\Lambda}\,\xi_{ij} - \boldsymbol{\Lambda}\,\boldsymbol{\kappa}_j + \boldsymbol{\varepsilon}_{ij}, \boldsymbol{\Lambda}\,\xi_{ij} - \boldsymbol{\Lambda}\,\boldsymbol{\kappa}_j + \boldsymbol{\varepsilon}_{ij})$$

$$= \mathrm{COV}(\boldsymbol{\Lambda}\,\xi_{ij} - \boldsymbol{\Lambda}\,\boldsymbol{\kappa}_j, \boldsymbol{\Lambda}\,\xi_{ij} - \boldsymbol{\Lambda}\,\boldsymbol{\kappa}_j) + 2\,\mathrm{COV}(\boldsymbol{\Lambda}\,\xi_{ij} - \boldsymbol{\Lambda}\,\boldsymbol{\kappa}_j, \boldsymbol{\varepsilon}_{ij}) + \mathrm{COV}(\boldsymbol{\varepsilon}_{ij}, \boldsymbol{\varepsilon}_{ij})$$

In factor analysis, residual factors are assumed to be independent of common factors, so the middle part is zero,

$$= \mathrm{COV}(\boldsymbol{\Lambda}\,\xi_{ij} - \boldsymbol{\Lambda}\,\boldsymbol{\kappa}_j, \boldsymbol{\Lambda}\,\xi_{ij} - \boldsymbol{\Lambda}\,\boldsymbol{\kappa}_j) + \mathrm{COV}(\boldsymbol{\varepsilon}_{ij}, \boldsymbol{\varepsilon}_{ij})$$

$\boldsymbol{\Lambda}$ is a constant and can go outside the brackets,

$$= \boldsymbol{\Lambda}\,\mathrm{COV}(\xi_{ij} - \boldsymbol{\kappa}_j, \xi_{ij} - \boldsymbol{\kappa}_j)\,\boldsymbol{\Lambda} + \mathrm{COV}(\boldsymbol{\varepsilon}_{ij}, \boldsymbol{\varepsilon}_{ij})$$

$\xi_{ij} - \boldsymbol{\kappa}_j$ are the individual factor deviation scores (in JOD these are incorrectly represented by ξ_{ij} itself), whose covariance matrix is denoted $\boldsymbol{\Phi}_W$, and $\mathrm{COV}(\boldsymbol{\varepsilon}_{ij}, \boldsymbol{\varepsilon}_{ij})$ is known as the residual (co)variance matrix $\boldsymbol{\Theta}_W$. So,

$$\boldsymbol{\Sigma}_W = \boldsymbol{\Lambda}\,\boldsymbol{\Phi}_W\,\boldsymbol{\Lambda} + \boldsymbol{\Theta}_W. \qquad (14)$$

The model-implied covariance matrix at the between level is:

$$\boldsymbol{\Sigma}_B = \mathrm{COV}(\boldsymbol{\mu}_j, \boldsymbol{\mu}_j), \qquad (15)$$

[1] One may rewrite this expression as $\mathbf{y}_{ij} - \boldsymbol{\mu}_j = \boldsymbol{\Lambda}(\xi_{ij} - \boldsymbol{\kappa}_j) + \boldsymbol{\varepsilon}_{ij}$, showing that with weak factorial invariance observed mean differences are a function of $\boldsymbol{\Lambda}$ times factor mean differences (and error).

$= \mathrm{COV}(\boldsymbol{\tau}_j + \boldsymbol{\Lambda}\, \boldsymbol{\kappa}_j , \boldsymbol{\tau}_j + \boldsymbol{\Lambda}\, \boldsymbol{\kappa}_j)$,

$= \mathrm{COV}(\boldsymbol{\Lambda}\, \boldsymbol{\kappa}_j , \boldsymbol{\Lambda}\, \boldsymbol{\kappa}_j) + 2\mathrm{COV}(\boldsymbol{\Lambda}\, \boldsymbol{\kappa}_j , \boldsymbol{\tau}_j) + \mathrm{COV}(\boldsymbol{\tau}_j , \boldsymbol{\tau}_j)$,

Assuming that the intercepts (residual means) are independent form the factor means, makes the middle part disappear:

$= \mathrm{COV}(\boldsymbol{\Lambda}\, \boldsymbol{\kappa}_j , \boldsymbol{\Lambda}\, \boldsymbol{\kappa}_j) + \mathrm{COV}(\boldsymbol{\tau}_j , \boldsymbol{\tau}_j)$,

$\boldsymbol{\Lambda}$ is a constant and can go outside the brackets,

$= \boldsymbol{\Lambda}\, \mathrm{COV}(\boldsymbol{\kappa}_j , \boldsymbol{\kappa}_j) \boldsymbol{\Lambda} + \mathrm{COV}(\boldsymbol{\tau}_j , \boldsymbol{\tau}_j)$,

If we call the covariance matrix of common factor means $\boldsymbol{\Phi}_B$, and the covariance matrix of intercepts $\boldsymbol{\Theta}_B$, then this becomes:

$$\boldsymbol{\Sigma}_B = \boldsymbol{\Lambda}\, \boldsymbol{\Phi}_B\, \boldsymbol{\Lambda} + \boldsymbol{\Theta}_B\, . \qquad (16)$$

Hence, if weak factorial invariance across groups holds, then the following two-level model holds:

$$\boldsymbol{\Sigma}_W = \boldsymbol{\Lambda}\, \boldsymbol{\Phi}_W\, \boldsymbol{\Lambda} + \boldsymbol{\Theta}_W$$

and

$$\boldsymbol{\Sigma}_B = \boldsymbol{\Lambda}\, \boldsymbol{\Phi}_B\, \boldsymbol{\Lambda} + \boldsymbol{\Theta}_B\, .$$

Assuming strong factorial invariance across clusters

If the values of factor loadings and intercepts are equal across groups, so that strong factorial invariance holds, then the model for the observed scores becomes:

$\mathbf{y}_{ij} = \boldsymbol{\tau} + \boldsymbol{\Lambda}\, \xi_{ij} + \boldsymbol{\varepsilon}_{ij}$ (no subscript to $\boldsymbol{\Lambda}$ and $\boldsymbol{\tau}$)

Compared with the situation with weak factorial invariance, equal intercepts across groups has no additional effect on $\boldsymbol{\Sigma}_W$ as the parameter $\boldsymbol{\tau}$ only features on the between-level. At the between-level, $\mathrm{COV}(\boldsymbol{\tau}_j , \boldsymbol{\tau}_j)$ from Equation 15 will become $\mathrm{COV}(\boldsymbol{\tau} , \boldsymbol{\tau})$, which is zero. Therefore, $\boldsymbol{\Theta}_B$ will be zero. Hence, if strong factorial invariance across groups holds, then the following two-level model holds:

$$\boldsymbol{\Sigma}_W = \boldsymbol{\Lambda}\, \boldsymbol{\Phi}_W\, \boldsymbol{\Lambda} + \boldsymbol{\Theta}_W \quad \text{and} \quad \boldsymbol{\Sigma}_B = \boldsymbol{\Lambda}\, \boldsymbol{\Phi}_B\, \boldsymbol{\Lambda}\, . \qquad (17)$$

Appendix B.

Mplus syntax for the final models. The example dataset is available from the first author upon request (S.Jak@uva.nl)

```
  TITEL: Measurement model
  DATA:
    FILE IS ESSwellbeing.dat;
  VARIABLE:
  NAMES ARE fltdpr wrhpp enjlf
             fltsd fltanx fltpcfl
             IL
              CPI
             cntryID;
    USEVARIABLES ARE fltdpr wrhpp enjlf fltsd fltanx fltpcfl;
    MISSING ARE fltdpr wrhpp enjlf fltsd fltanx fltpcfl (999);

  MODEL:       posWB by wrhpp;
               posWB by enjlf;
               posWB by fltpcfl;

               negWB by fltdpr;
               negWB by fltsd;
               negWB by fltanx;
               negWB by fltpcfl; ! This is the added cross loading
```

```
TITEL: Multigroup models
  DATA:
  FILE IS ESSwellbeing.dat;
  VARIABLE:
  NAMES ARE fltdpr wrhpp enjlf
                  fltsd fltanx fltpcfl
                  IL
                  CPI
                  cntryID;
    USEVARIABLES ARE fltdpr wrhpp enjlf fltsd fltanx fltpcfl;
    MISSING ARE fltdpr wrhpp enjlf fltsd fltanx fltpcfl (999);
    GROUPING IS cntryID (1=AL 2=BE 3=BG 4=CH 5=CY 6=CZ 7=DE 8=DK
                      9=EE 10=ES 11=FI 12=FR 13=GB 14=HU
                       15=IE 16=IL 17=IS 18=IT 19=LT 20=NL
                       21=NO 22=PL 23=PT 24=RU 25=SE 26=SI
                       27=SK 28=UA 29=XK);
  ANALYSIS: MODEL IS CONFIGURAL METRIC SCALAR;
  MODEL:       posWB by wrhpp;
               posWB by enjlf;
               posWB by fltpcfl;

               negWB by fltdpr;
               negWB by fltsd;
               negWB by fltanx;
               negWB by fltpcfl;
```

```
TITEL: Final two-level model
  DATA:
    FILE IS ESSwellbeing.dat;
  VARIABLE:
  NAMES ARE fltdpr wrhpp enjlf
            fltsd fltanx fltpcfl
            IL
            CPI
```

```
            cntryID;
    USEVARIABLES ARE fltdpr wrhpp enjlf fltsd fltanx fltpcfl;
    MISSING ARE fltdpr wrhpp enjlf fltsd fltanx fltpcfl (999);
    Cluster IS cntryID;

ANALYSIS: TYPE IS twolevel;
MODEL:
       %WITHIN%
            posWB by wrhpp* enjlf fltpcfl (l1-l3);
            posWB@1;

            negWB by fltdpr* fltsd fltanx fltpcfl (l4-l7);
            negWB@1;
            fltdpr-fltpcfl (tw1-tw6);

      %BETWEEN%
            cposWB by wrhpp* enjlf fltpcfl (l1-l3);
            cposWB (PHIb_POS);

            cnegWB by fltdpr* fltsd fltanx fltpcfl (l4-l7);
            cnegWB (PHIb_NEG);

            fltdpr-fltpcfl (tb1-tb6);
```
